# Rehabilitation strategy for hip fracture, focused on behavioral psychological symptoms of dementia for older people with cognitive impairment: A nationwide Japan rehabilitation database

**DOI:** 10.1371/journal.pone.0200143

**Published:** 2018-07-05

**Authors:** Koji Shibasaki, Toshiomi Asahi, Keiko Mizobuchi, Masahiro Akishita, Sumito Ogawa

**Affiliations:** 1 Department of Geriatric Medicine, Graduate School of Medicine, The University of Tokyo, Bunkyo-ku, Tokyo, Japan; 2 Asahi Neurology and Rehabilitation Hospital, Matsudo City, Chiba, Japan; Ehime University Graduate School of Medicine, JAPAN

## Abstract

The aim is to investigate the relationship between a positive outcome on rehabilitation after hip fracture and behavioral psychological symptoms of dementia (BPSD) transition during rehabilitation. This study is a retrospective cohort study based on the Japan Rehabilitation Database. We recruited 756 subjects 65 years of age or older from 31 hospitals in the database. All subjects were in the hospital as patients undergoing rehabilitation for hip fracture. Functional independence measure (FIM), walking ability, Mini-Mental State Examination (MMSE), and BPSD were measured both at the beginning and at the end of rehabilitation. MMSE for 23 or under was defined as the cognitive-impaired group. MMSE for 24 or over was used as the cognitively intact group. Cognitive impaired participants were divided into four groups: participants presented no BPSD both at the beginning of rehabilitation and at the end of rehabilitation (Group (-/-)), participants presented BPSD at the beginning of rehabilitation but resolved at the end of rehabilitation (Group (+/-)), participants had no BPSD at the beginning of rehabilitation but appeared at the end of rehabilitation (Group (-/+)) and participants had sign of BPSD both at the beginning of rehabilitation and at the end of rehabilitation (Group (+/+)). The endpoints were waking ability, FIM gain. As results, one hundred thirty-seven cognitive-impaired older people patients out of 471 (29.1%) suffered from BPSD at the beginning of rehabilitation. FIM gains in cognitively intact group, Group (-/-), Group (+/-), Group (-/+) and Group (+/+) were 24.8 ± 18.7, 17.5 ± 16.9, 27.3 ± 19.7, 17.8 ± 12.2 and 12.2 ± 17.2, respectively. The Group (+/-) was significantly connected to a positive outcome for rehabilitation. The present study suggested that the management of BPSD can lead to better functional recovery during rehabilitation.

## Introduction

Thus far, many studies have demonstrated that cognitive impairment has been one of the major factors that limit functional recovery during rehabilitation for older people with hip fracture. However, few reports focus on how rehabilitation of older people with dementia is handled. [1] Hip fracture is one of the major causes of low functional capacity in older people. The risk for those with cognitive impairment is two to seven times higher than for those without cognitive impairment. [2–7] In addition, 20% to 30% of older people with hip fracture suffer from cognitive impairment. [8–10] Many studies have shown that when older people with cognitive impairment suffered hip fracture, functional recovery during rehabilitation was limited compared to those whose cognition remained intact. This fact has been confirmed by numerous large-scale clinical studies, systematic reviews, and meta-analyses. [11–18]

Older people with cognitive impairment who fracture their hip have a greater risk of comorbidity such as delirium, [19] pressure ulcer, surgical site infection, [20, 21] urinary tract infection, and respiratory infection. [20] Furthermore, the pain score was higher in cognitive-impaired older people with hip fracture than in cognitive-intact older people. [22] The rate of falls [18] and mortality [11, 13, 23–25] was also higher in cognitive-impaired older people after hip surgery.

Although rehabilitation has been reported to improve physical function for the cognitive-impaired older people with hip fracture, [13, 26–28] the chance to be in a rehabilitation unit is significantly lower for the cognitive-impaired older people than for the cognitive-intact older people. [2, 13, 24] Some studies have researched the efficacy of rehabilitation for the cognitive-impaired older people with hip fracture. In those articles, rehabilitation for the cognitive-impaired older people with hip fracture improved not only physical function but also quality of life, care burden, and mortality. [27, 29]

The number of cognitive-impaired older people is increasing in the world [30]; however, few studies have mentioned the management of rehabilitation for older people with cognitive impairment. As far as we know, only multidisciplinary geriatric assessment has been connected with the beneficial effect after hip fracture for older people with cognitive impairment. [1] Therefore, we investigated the strategy of rehabilitation for older people with cognitive impairment.

Because psychological symptoms such as delirium and depression prevent older people from functional recovery after hip fracture, we focused on psychological symptoms during rehabilitation. The aim is to investigate the relationship between a positive outcome of rehabilitation after hip fracture and behavioral psychological symptoms of dementia (BPSD) transition during rehabilitation. We researched whether improvement of BPSD might lead to a beneficial outcome of rehabilitation for older people with hip fracture.

## Materials and methods

### Japan rehabilitation database

Detailed data was collected in the Japan Association of Rehabilitation Database from 2005 to 2015. The data set is separated by three categories: hip fracture, cerebrovascular disease, and spinal cord injury. The present study used the database of hip fracture. It includes the following data: age, sex, physical function (walking ability, functional independence measure [FIM], and Barthel index), cognitive function, days from injury onset, type of fracture, information about long-term insurance, length of stay, BPSD, comorbidities, past history, and other detailed data that affects rehabilitation.

All personal data was coded, deleting any information related to personal identification. Patients did not provide informed consent for inclusion of their data in the database and subsequent use of the data in research investigations. Informed consent was waived because of the anonymous nature of the data. [31] This study protocol was approved by the institutional review board of the Japan Association of Rehabilitation Medicine. Ethical approval had previously been granted by the Japanese Association of Rehabilitation Medicine; the reference is available on the Japan Association of Rehabilitation Database website. [32]

### Participants

**Fig 1:**
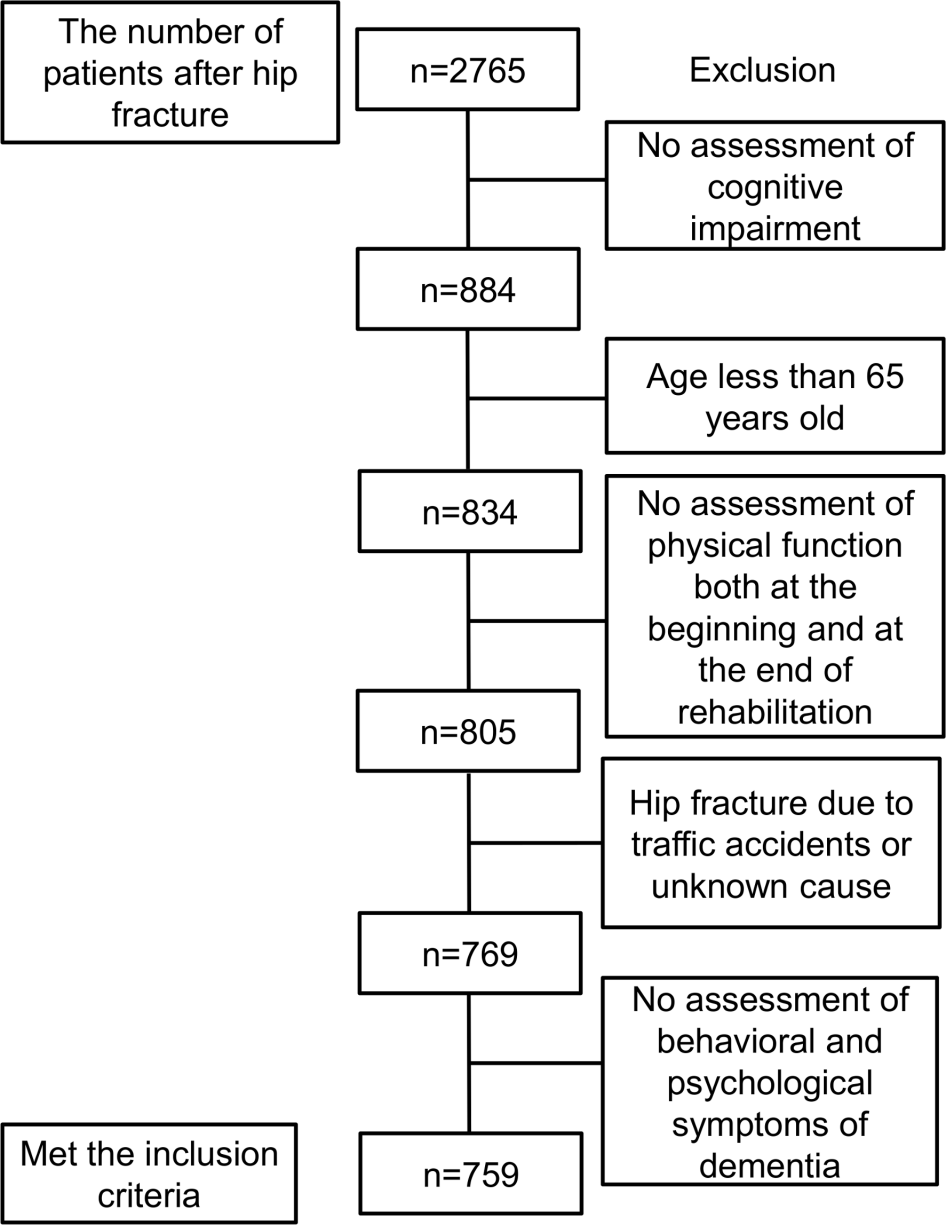
Flow chart of eligibility for the database after hip fracture.

The inclusion and exclusion criteria of the study are shown in Fig 1. Of 2765 older people, we included those aged 65 or older, and we evaluated physical function, cognitive function, BPSD, and walking ability both at the beginning of and at the end of rehabilitation, and fracture as a result of fall. We excluded the participants who received a fracture from a traffic accident or an unknown cause.

### Measurement and outcomes

The data of age, sex, comorbidities (cerebrovascular disease, neurodegenerative disease, osteoarthritis, and past history of fracture), cognitive function, physical function, walking ability, BPSD, and length of stay were obtained from the Japan Rehabilitation database. Cognitive function and physical function were evaluated by the Mini-Mental State Examination (MMSE) and FIM, respectively. MMSE was evaluated three times in the database after operation, at the beginning of rehabilitation and at the end of rehabilitation. To minimize the effect of operation itself and operation-related psychological effect, we used maximum MMSE score among these three evaluations. The data classified walking ability in the following categories: walking independently, walking with cane or walker, and cannot walk. MMSE for 23 or under was defined as the cognitive-impaired group. MMSE for 24 or over was used as the cognitively intact group. BPSD was defined as presenting the following symptoms: delirium, delusion, wandering, insomnia, depression, aggression, and violence. Because BPSD was evaluated as only BPSD positive (+) or BPSD negative (-), the sign and symptom from which patients were suffering was unclear.

Outcomes were assessed by FIM gain (FIM at the end of rehabilitation–FIM at the beginning of rehabilitation) and FIM gain per day (FIM gain/length of stay). FIM contains 18 items. Thirteen items pertain to motor function (motor FIM), five items concern cognitive function (cognitive FIM). Motor FIM gain, motor FIM gain per day, cognitive FIM, and cognitive FIM per day were also assessed as outcomes.

### Intervention (rehabilitation)

The typical rehabilitation program was conducted for all patients. It was composed of 40 minutes of physical therapy and 40 minutes of occupational therapy per day, for 5 to 7 days per week. Physical and occupational therapy focused on gait and exercise related to activities of daily living. Passive range-of-motion exercise of the affected side and muscle-strengthening exercise of the unaffected side were included. [31]

### Statistical analysis

Data were analyzed using SPSS software (v. 23, SPSS Japan Inc., Japan). Two-sided unpaired *t*-test for continuous variables and chi-squared test for categorical variables were used to compare cognitively intact and older people with cognitive impairment. After older people with cognitive impairment group was classified into four groups according to BPSD transition, we investigated the difference of functional recovery among subgroups divided by BPSD transition. The cognitive-impaired group was divided into four groups according to BPSD present (+) or absent (-) both at the beginning and the end of rehabilitation. We evaluated the change of MMSE (MMSE at the end of rehabilitation–MMSE at the beginning of rehabilitation) based on the previous study. [33] Analysis of variance (ANOVA) was used for comparison between the cognitively intact and older people with cognitive impairment subgroups. The Bonferroni method was used for post hoc tests after ANOVA. Two multinomial logistic regression analyses were built. The first was a crude model. Adjusted model adjusted for age, sex, comorbidities (cardiovascular disease, neurodegenerative disease, osteoarthritis, and past history of fracture), walking ability before fracture, FIM at the beginning of rehabilitation, MMSE and the length of hospital stay. The chi-squared test also computed a comparison of the walking ability both at the beginning of and at the end of rehabilitation.

## Results

We recruited 759 of older people from the Japan Rehabilitation database (Fig 1). The number of patients in the cognitively intact group and cognitive-impaired group were 288 and 471, respectively (Table 1). The background data is shown in Table 1. Age, prevalence of cerebrovascular disease, walking ability, MMSE, and FIM were significantly lower in the cognitive-impaired group than in the cognitively intact group. The length of rehabilitation time in physical therapy, occupational therapy, and speech therapy were 46.6 ± 28.1, 28.1 ± 14.3, and 4.1 ± 6.2 minutes per day, respectively.

**Table 1:** Characteristics of older individuals.

Variable	Subgroups	Cognitively intact group	Cognitive-impaired group	p-value
**Number of Subjects**		288	471	
**Age**		81.6±7.4	85.4±6.9	<0.001
**Sex, male (%)**		61/288 (21.2)	90/471 (19.1)	0.488
**Comorbidity, (%)**	Cerebrovascular disease	35 (12.2)	86 (18.3)	0.026
	Neurodegenerative disease	6 (2.1)	13 (2.8)	0.563
	Osteoarthritis	66 (22.9)	103 (21.9)	0.736
	Fracture	68 (23.6)	120 (25.5)	0.563
**Walking ability before fracture, (%)**	Walk independently	183 (63.5)	195 (41.4)	<0.001
	Walk with cane or walker	96 (33.3)	220 (46.7)	
	Cannot walk	6 (2.1)	52 (11.0)	
	Unknown	3 (1.0)	4 (0.8)	
**MMSE**		27.4±2.1	14.3±6.6	<0.001
**FIM at the beginning of rehabilitation**		80.9±23.2	51.6±24.9	<0.001

Values are mean ± SD. MMSE, Mini-Mental State Examination; FIM, functional independence measure.

The length of stay was no different between the cognitively intact and cognitive-impaired groups (45.0 ± 29.7 vs. 46.0 ± 27.0, respectively) (Table 2). However, FIM, FIM gain, FIM gain per day, and walking ability were significantly lower in the cognitive-impaired group than in the cognitively intact group at the end of rehabilitation.

**Table 2:** Effect of rehabilitation.

Variable	Subgroups	Cognitively intact group	Cognitive impaired-group	p-value
**Length of stay**		46.0±27.0	45.0±29.7	0.262
**FIM at the end of rehabilitation**		105.8±19.6	68.8±26.8	<0.001
**FIM gain**		24.8±18.7	17.1±17.6	<0.001
**FIM gain per day**		0.78±0.78	0.50±0.70	<0.001
**Walking ability after rehabilitation, (%)**	Walk independently	61(21.2)	20 (4.2)	<0.001
	Walk with cane or walker	201(69.8)	266 (56.5)	
	Cannot walk	23 (8.0)	182 (38.6)	
	Unknown	3 (1.0)	3 (0.6)	

Values are mean ± SD. FIM, functional independence measure.

Next, we investigated the difference of functional recovery among subgroups divided by BPSD transition (Table 3). The cognitive-impaired group was divided into four groups according to BPSD present (+) or absent (-) both at the beginning and end of rehabilitation. Three hundred and twenty-six patients were BPSD (-) both at the beginning and end of rehabilitation (Group (-/-)). Forty-one subjects were BPSD (+) at the beginning and BPSD (-) at the end of rehabilitation (Group (+/-)). Eight subjects were BPSD (-) at the beginning and BPSD (+) at the end of rehabilitation (Group (-/+)). Ninety-six patients were BPSD (+) both at the beginning and end of rehabilitation (Group (+/+)). The occurrence of BPSD at the beginning of rehabilitation was noted in 137 subjects (Group (+/-) and Group (+/+), 29.1%) out of 471 subjects in the cognitive-impaired group. In those with BPSD, 41 subjects (Group (+/-)) out of 137 subjects had diminished BPSD at the end of rehabilitation. Ninety-six (Group (+/+)) continued with BPSD during rehabilitation. The distribution of background data was not statistically different among the four groups except for MMSE and FIM. MMSE and FIM at the beginning of rehabilitation were significantly lower in Group (+/+). The change of MMSE were -0.24 ± 6.4, -0.57 ± 5.2, 3.00 ± 5.4, -0.55 ± 7.5 and 0.78 ± 4.3 in cognitively intact, Group (-/-), Group (-/+), Group (+/-) and Group (+/+), respectively. ANOVA and Bonferroni method revealed no significant difference among five groups.

**Table 3:** Characteristics of older individuals among subgroups divided by behavioral psychological symptoms of dementia transition.

Variable				
Subgroups	Group (-/-)	Group (+/-)	Group (-/+)	Group (+/+)
**BPSD at the beginning of rehabilitation**	-	+	-	+
**BPSD at the end of rehabilitation**	-	-	+	+
**Number of subjects (%)**	326 (69.2)	41 (8.7)	8 (1.7)	96 (20.4)
**Age**	85.1±6.9	87.5±5.6	85.9±5.6	85.2±7.3
**Sex, male (%)**	60(18.4%)	7(17.1%)	2(25.0%)	21(21.9%)
**MMSE**	15.3±6.4	14.8±6.1	13.1±6.1	10.9±6.4^†^*
**FIM at the beginning of rehabilitation**	55.5±26.0	45.5±20.2	47.0±21.6	41.5±19.2*
**Length of stay, days**	44.9±28.5	39.3±29.4	42.0±32.0	48.0±33.5

Values are mean ± SD. BPSD, behavioral psychological symptoms of dementia; FIM, functional independence measure; MMSE, Mini-Mental State Examination.

*p<0.05 vs. Group (-/-)

^†^p<0.05 vs. Group (+/-).

**Fig 2:**
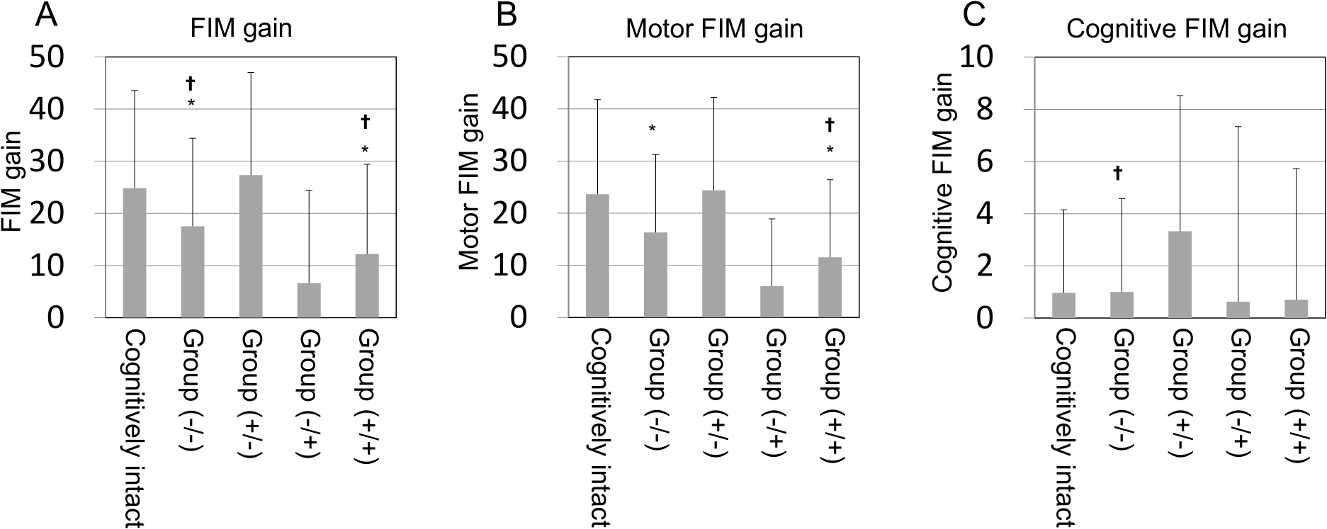
FIM gain among cognitively intact and cognitive impaired groups divided by BPSD. FIM, functional independence measure; data is mean ± standard deviation. Group (-/-): BPSD (-) both at the beginning of and at the end of rehabilitation. Group (+/-): BPSD (+) at the beginning and BPSD (-) at the end of rehabilitation. Group (-/+): BPSD (-) at the beginning of rehabilitation and BPSD (+) at the end of it. Group (+/+): BPSD (+) both at the beginning of and at the end of rehabilitation. Analyzed by ANOVA and Bonferroni as a post-hoc test. (A) FIM gain. (B) motor FIM gain. (C) cognitive FIM gain. * vs. cognitively intact; ✝ vs. Group (+/-).

FIM gain in the cognitively intact group, Group (-/-), Group (+/-), Group (-/+), and Group (+/+) was 24.8 ± 18.7, 17.5 ± 16.9, 27.3 ± 19.7, 6.6 ± 17.8, and 12.2 ± 17.2, respectively (Fig 2A). FIM gain per day in these groups was 0.78 ± 0.78, 0.53 ± 0.68, 0.94 ± 0.81, 0.03 ± 1.25, and 0.28 ± 0.54, respectively. Regarding FIM gain and FIM gain per day, Group (+/-) improved equal to the cognitively intact group. Group (-/-), Group (-/+), and Group (+/+) significantly diminished in both FIM gain and FIM gain per day compared with the cognitively intact group and Group (+/-) (FIM gain: ANOVA, F [4,754] = 14.350, p <0.001, FIM gain per day: ANOVA, F [4,754] = 13.685, p <0.001). FIM gain per day in Group (+/+) was significantly lower than FIM gain per day in Group (-/-). Motor FIM gain in these groups was 23.6 ± 18.1, 16.3 ± 14.9, 24.4 ± 17.8, 6.0 ± 12.9, and 11.6 ± 14.9, respectively (ANOVA, F (4,717) = 14.696, p <0.001) (Fig 2B). Motor FIM gain per day in these groups was 0.74 ± 0.77, 0.48 ± 0.59, 0.84 ± 0.73, 0.11 ± 1.17, and 0.27 ± 0.47, respectively (ANOVA, F (4,717) = 12.770, p <0.001). Cognitive FIM at the beginning of rehabilitation in Group (-/-), Group (+/-), Group (+/-) and Group (+/+) were 20.2±8.3, 16.6±7.8, 15.9±5.4, 13.4±6.1, respectively. Cognitive FIM at the end of rehabilitation in Group (-/-), Group (+/-), Group (+/-) and Group (+/+) were 21.2±8.0, 19.9±7.5, 16.5±7.3, 14.1±5.3, respectively. Cognitive FIM gain in these groups was 0.96 ± 3.18, 1.00 ± 3.58, 3.33 ± 5.20, 0.63 ± 6.72, and 0.70 ± 5.03, respectively (ANOVA, F (4,741) = 3.835, p = 0.004) (Fig 2C). Cognitive FIM gain per day in these groups was 0.02 ± 0.08, 0.03 ± 0.14, 0.10 ± 0.21, –0.08 ± 0.20, and 0.01 ± 0.19, respectively (ANOVA, F (4,741) = 4.989, p = 0.001). All results were similar to the results of FIM gain and FIM gain per day. In addition, cognitive FIM gain per day was significantly higher in Group (+/-) than in the cognitively intact group. Furthermore, multinomial logistic regression analysis confirmed these associations (Table 4). Group (+/-) significantly improved, cognitive FIM gain compared with Group (-/-), and all three rehabilitation outcomes were significantly improved in Group (+/-) compared to Group (-/+) and Group (+/+).

**Table 4:** Odds ratios (OR) (and 95% CIs) of rehabilitation outcomes obtained from multinomial logistic regression analyses among cognitively intact and cognitive-impaired groups with or without behavioral psychological symptoms of dementia.

		Crude	Adjusted model		Crude	Adjusted model		Crude	Adjusted model		Crude	Adjusted model
	vs cognitively intact	OR	OR	vs Group (+/-)	OR	OR	vs Group (-/-)	OR	OR	vs Group (-/+)	OR	OR
FIM gain	Group (+/-)	1.01 (0.99, 1.03)	1.02 (1.00, 1.04)									
Motor FIM gain		1.00 (0.98, 1.02)	1.02 (0.99, 1.04)									
Cognitive FIM gain		1.14 (1.06, 1.23)**	1.14 (1.05, 1.24)**									
FIM gain	Group (-/-)	0.98 (0.97, 0.99)**	0.99 (0.98, 1.01)	Group (-/-)	0.98 (0.96, 0.99)**	0.98 (0.97, 1.00)						
Motor FIM gain		0.97 (0.96, 0.98)**	1.00 (0.98, 1.01)		0.98 (0.97, 1.00)*	0.99 (0.97, 1.01)						
Cognitive FIM gain		1.00 (0.96, 1.05)	0.99 (0.93, 1.04)		0.88 (0.82, 0.93)**	0.87 (0.81, 0.93)**						
FIM gain	Group (-/+)	0.94 (0.90, 0.98)**	0.96 (0.93, 1.00)*	Group (-/+)	0.93 (0.89, 0.96)**	0.93 (0.90, 0.97)**	Group (-/+)	0.96 (0.92, 1.00)	0.96 (0.92, 1.00)*			
Motor FIM gain		0.93 (0.89, 0.97)**	0.96 (0.92, 1.00)		0.92 (0.88, 0.96)**	0.94 (0.90, 0.98)**		0.95 (0.91, 1.00)*	0.95 (0.91, 1.00)*			
Cognitive FIM gain		0.98 (0.81, 1.18)	0.92 (0.80, 1.07)		0.80 (0.68, 0.94)**	0.76 (0.66, 0.88)**		0.98 (0.82, 1.16)	0.92 (0.80, 1.06)			
FIM gain	Group (+/+)	0.96 (0.95, 0.97)**	0.97 (0.96, 0.99)**	Group (+/+)	0.95 (0.93, 0.97)**	0.95 (0.93, 0.97)**	Group (+/+)	0.98 (0.97, 1.00)**	0.98 (0.96, 0.99)**	Group (+/+)	1.02 (0.98, 1.06)	1.00 (0.93, 1.07)
Motor FIM gain		0.95 (0.94, 0.97)**	0.97 (0.96, 0.99)**		0.95 (0.93, 0.97)**	0.96 (0.94, 0.98)**		0.98 (0.96, 0.99)**	0.98 (0.96, 0.99)**		1.03 (0.98, 1.08)	1.04 (0.94, 1.15)
Cognitive FIM gain		0.98 (0.92, 1.04)	0.95 (0.88, 1.01)		0.85 (0.78, 0.92)**	0.81 (0.75, 0.88)**		0.98 (0.93, 1.04)	0.95 (0.89, 1.01)		1.00 (0.87, 1.15)	0.93 (0.80, 1.09)

High odds ratios indicate higher at each FIM gain (favorable rehabilitation outcome), and low odds ratios indicate lower at each FIM gain (unfavorable rehabilitation outcome). Adjusted model was adjusted for age, sex, comorbidities (cerebrovascular disease, neurodegenerative disease, osteoarthritis, and past history of fracture), walking ability before fracture, baseline functional independence measure at the beginning of rehabilitation, mini-mental state examination and the length of hospital stay. FIM, functional independence measure.

*p <0.05

**p <0.01

While BPSD was significantly associated with rehabilitation outcomes independent from MMSE, MMSE was also independent predictive factor for rehabilitation outcomes.

**Fig 3:**
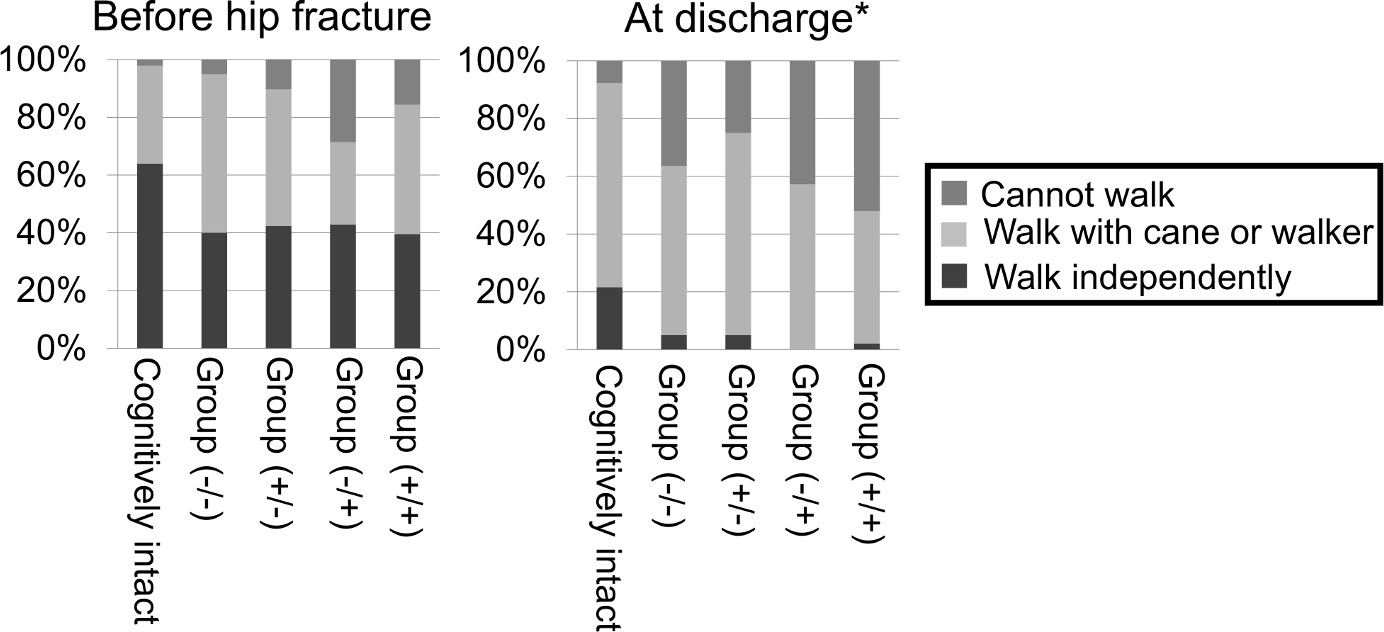
Transition of walking ability. *p<0.001 Chi-squared test among subgroups.

The rate of individuals who could walk independently was higher in the cognitively intact group than in the cognitive-impaired group before hip fracture. Conversely, the rate of individuals who needed a cane or walker for walking or who could not walk was higher in the cognitive-impaired group. Similar results were detected at the end of rehabilitation. Walking ability was significantly higher in the cognitively intact group than in the cognitive-impaired group (Tables 2 and 3). When the cognitive-impaired group was divided into four groups according to BPSD transition, even though the walking ability before hip fracture was not statistically significant (p = 0.052), the rate of older adults who could walk either independently or with cane/walker was higher in Group (+/-) among the four groups at the end of rehabilitation (p <0.001) (Fig 3).

## Discussion

The present study showed that older people with cognitive impairment recovered less of their physical function during rehabilitation than older people without cognitive impairment. The prevalence of BPSD was 29.1% (137/471), and BPSD had disappeared in 41 (29.9%) of 137 individuals. Interestingly, BPSD was an important predictive factor of rehabilitation outcome for older people with hip fracture and cognitive impairment. Our study also suggests that the recovery from BPSD might lead to positive outcomes, such as improvement of waking ability and FIM gain, during rehabilitation.

Stenvall M et al. illustrated a multidisciplinary intervention program, such as comprehensive geriatric assessments and rehabilitation, designed to prevent delirium, falls, decubital ulcers, and malnutrition, which improved rehabilitation outcome after hip fracture for older people with dementia. [1] Our study corresponds to the study Stenvall M et al. reported, because both studies focused on psychological symptoms such as delirium. These two studies demonstrated the importance of comprehensive geriatric assessments.

In our data, the number of patients who presented BPSD at the beginning of rehabilitation was 137 (29.1% in Group (+/-) plus Group (+/+)) of 471 cognitive-impaired patients. Givens et al. measured the prevalence of depressive symptoms, cognitive impairment, and delirium in patients with hip fracture and found that 22% of patients had one cognitive or mood disorder, 30% had two, and 7% had three. [15] Other studies have reported that the prevalence of anxiety or mood disorder was 35% to 36%. The prevalence of BPSD in our data was similar to the present study. Forty-one patients’ BPSD resolved (29.9%, Group (+/-)) out of 137 (Group (+/-) and Group (+/+)). To compare the background data between Group (+/-) and Group (+/+), MMSE was higher in Group (+/-) than in Group (+/+), but it was not statistically significant. Several studies demonstrated that functional outcome during rehabilitation after hip surgery was no different between mild to moderate cognitive impairment and the cognitive-intact group, although functional recovery during rehabilitation was impeded in older people with severe cognitive impairment. [14, 16, 17, 34] Our data suggested the possibility that BPSD is an important impediment to functional recovery in the severe cognitive-impairment group, because the prevalence of BPSD in older people with severe cognitive impairment was higher than in older people with mild to moderate cognitive impairment. Furthermore, BPSD was less relieved in the severe cognitive-impaired group. Whereas previous studies have demonstrated that cognitive impairment diminished the rehabilitation outcome, our multinomial regression analyses showed that BPSD was an independent predictive factor for rehabilitation outcome after adjusted for MMSE. And our result showed no significant difference in the change of cognitive function among 4 subgroups, which was in contrast to previous study that MMSE change was associated with rehabilitation outcome.

Our study could not show which symptoms were present in the BPSD-positive group, but it has been reported in other studies that agitation was the most frequent symptom, followed by depression, apathy, and other behavioral disorders in patients with Alzheimer’s disease or multi-infarct dementia. [35, 36] The reason for the high incidence of BPSD after hip fracture was reported to be that hip surgery or hip fracture per se were major risk factors for the onset of BPSD. [5, 37] A diathesis-stress model was also argued to describe best the etiological pathway of hopelessness to depression and has potential application to a hip fracture population. [38]

Regarding the therapeutic intervention for BPSD, non-pharmacological treatment approaches have become the preferred first-line option. [39–41] When non-pharmacological treatment is not effective, pharmacological treatment is considered. [39, 41–43] As effective nonpharmacological interventions, person-centered care, communication skills training, adapted dementia care mapping, and music therapy [43–45] for agitation and aggression, music therapy, [46] and multisensory stimulations for apathy and depression were reported. Environmental approaches and behavioral approaches are also effective for BPSD. [45, 47, 48]

Pharmacological treatment such as with quetiapine, benzodiazepines, or anti-psychotic drugs for agitation and aggression, and antidepressant drugs for depression and apathy, were reported. [36, 40] However, pharmacological treatment entails adverse effects for older people. A small case-control study reported that the administration of anti-psychological drugs did not affect rehabilitation outcome [12, 49]; however, it did demonstrate that the administration of anti-psychological drugs was connected to falls, fractures, and increased mortality. [41, 50] The administration of anti-psychological drugs should be assessed carefully.

There are several limitations in the present study. First, this database did not evaluate which type of BPSD occurred. BPSD was defined in the database as delirium, delusion, wandering, insomnia, depression, aggression, and violence. However, each sign and symptom should be evaluated individually. Second, therapeutic intervention was not clear. Rehabilitation or exercise intervention was reported to reduce the prevalence of BPSD. [29, 51, 52] However, it is not clear whether only rehabilitation exercise intervention was planned or other non-pharmacological or pharmacological intervention was carried out. Third, this study is based on the rehabilitation database. A randomized-control study for intervention against BPSD should be required to confirm these results. Fourth, there is a possibility that cognitive function before hip fracture might influence rehabilitation outcomes. Unfortunately, the database we used in this study did not contain cognitive function before hip fracture. In the previous study, there was only an article which described about cognitive function before hip fracture surgery instead of hip fracture itself [53]. In the near future, influence of cognitive function before hip fracture on rehabilitation outcomes would be clarified based on prospective cohort studies. Finally, we excluded 2006 participants because of missing data. The present study may not represent the hip fracture population.

To the best of our knowledge, allowing these limitations, only a few studies showed positive outcomes of in-patient rehabilitation for older people with cognitive impairment after hip surgery. [1] Up until now, cognitive impairment for older people with hip fracture was reported to lead to a negative impact on rehabilitation. Previous studies have revealed that older people with cognitive impairment evince delirium, [19] pressure ulcer, surgical site infection, and other infections such as a urinary tract infection and infections of the respiratory system. [20, 21] To be sure, cognitive impairment is one of the most negative predictive factors for rehabilitation. However, few studies mention the positive factors or the precaution of negative factors on rehabilitation despite the high prevalence of cognitive impairment, dementia, and hip fracture in the world. [30] As far as we know, only multidisciplinary intervention was related to the positive impact on rehabilitation for older people with cognitive impairment. [1]

## Conclusion

The present study suggested that BPSD management has the possibility to lead to better functional recovery during rehabilitation. A positive outcome might be expected to assess the psychological symptoms and comorbidities that occur most often in older people with cognitive impairment.
